# A Rapid Assessment of Avoidable Blindness in Southern Zambia

**DOI:** 10.1371/journal.pone.0038483

**Published:** 2012-06-21

**Authors:** Robert Lindfield, Ulla Griffiths, Fiammetta Bozzani, Musonda Mumba, Joseph Munsanje

**Affiliations:** 1 International Centre for Eye Health, London School of Hygiene and Tropical Medicine (LSHTM), London, United Kingdom; 2 Department of Global Health and Development, London School of Hygiene and Tropical Medicine (LSHTM), London, United Kingdom; 3 Lusaka Eye Hospital, Lusaka, Zambia; 4 S & S Development Consultants, Lusaka, Zambia; Medical University Graz, Austria

## Abstract

**Introduction:**

A rapid assessment of avoidable blindness (RAAB) was conducted in Southern Zambia to establish the prevalence and causes of blindness in order to plan effective services and advocate for support for eye care to achieve the goals of VISION 2020: the right to sight.

**Methods:**

Cluster randomisation was used to select villages in the survey area. These were further subdivided into segments. One segment was selected randomly and a survey team moved from house to house examining everyone over the age of 50 years. Each individual received a visual acuity assessment and simple ocular examination. Data was recorded on a standard proforma and entered into an established software programme for analysis.

**Results:**

2.29% of people over the age of 50 were found to be blind (VA <3/60 in the better eye with available correction). The major cause of blindness was cataract (47.2%) with posterior segment disease being the next main cause (18.8%). 113 eyes had received cataract surgery with 30.1% having a poor outcome (VA <6/60) following surgery. Cataract surgical coverage showed that men (72%) received more surgery than women (65%).

**Discussion:**

The results from the RAAB survey in Zambia were very similar to the results from a similar survey in Malawi, where the main cause of blindness was cataract but posterior segment disease was also a significant contributor. Blindness in this part of Zambia is mainly avoidable and there is a need for comprehensive eye care services that can address both cataract and posterior segment disease in the population if the aim of VISION 2020 is to be achieved. Services should focus on quality and gender equity of cataract surgery.

## Introduction

Approximately 39 million people are blind globally [1]. Over 90% of those who are blind reside in low or middle income countries. Causes of blindness can be divided into avoidable (preventable or treatable) and non-avoidable [2].

VISION 2020 is a global initiative to eliminate avoidable blindness by the year 2020 [2]. It is led by a consortium of international non-government organisations under an umbrella organisation, the International Agency for the Prevention of Blindness (IAPB) in partnership with the World Health Organization (WHO). VISION 2020 emphasises the importance of understanding both the burden and causes of blindness as a critical part of planning robust eye care programmes [3].

Several surveys have been conducted to describe the prevalence and causes of blindness in sub-Saharan Africa[4]–[7]. The rapid assessment of avoidable blindness (RAAB) survey technique was developed as an efficient and quick way to describe blindness at district level (populations of approximately 1–2 million people) [8]. RAAB is a cross-sectional community-based survey aimed at assessing the prevalence of avoidable eye conditions and the coverage and outcomes of cataract surgery in a population above 50 years of age. The choice of an age group with the highest expected prevalence minimizes sample size requirements and ensures RAAB surveys are rapid and relatively cheap [8].

Zambia is a country in southern Africa of approximately 13 million people. It ranks as 164 out of 187 countries in the human development index (a marker of poverty) [9].

No information is available about the prevalence or causes of blindness in Zambia. However a previous estimate based on studies in other African countries suggested the prevalence of blindness for all age groups in the region is around 1%[10]–[11].The Zambian Government in partnership with Sightsavers International, an international non-governmental organisation, and Seeing is Believing, a funding collaboration between Standard Chartered Bank and IAPB, established a project in southern Zambia called The Livingstone to Lusaka Comprehensive Urban Eye Care Project that aims to reduce avoidable blindness. As part of this project a RAAB survey was conducted in the project area to establish baseline information about the prevalence and causes of blindness.

## Results

3,629 people were examined (response rate of 94.9%). 60.5% were women. 148 individuals (3.9%) were unavailable, 33 (0.9%) refused and 9 (0.2%) were not capable of taking part in the survey.

The survey took place in 80 clusters in eight districts within two provinces (Southern and Lusaka) in Southern Zambia. Table 1 shows the total population by age and gender of the survey area compared to the sample. The distribution of the population sampled is broadly similar to the population of the survey area.

**Table 1:** Age and gender distribution of the sample compared to the total population in the survey area.

AgeGroup	Male		Female		Total	
	Sample	Population	Sample	Population	Sample	Population
**50-54**	24.4%	26.8%	29.5%	30.5%	27.5%	28.5%
**55-59**	17.4%	19.7%	17.4%	20.9%	17.4%	20.3%
**60-64**	16.8%	17.3%	16.0%	19.8%	16.3%	18.5%
**65-69**	13.5%	14.1%	12.2%	13.5%	12.7%	13.9%
**70-74**	12.1%	9.8%	9.7%	8.9%	10.7%	9.4%
**75-79**	6.4%	6.1%	7.3%	0.5%	6.9%	3.5%
**80+**	9.3%	6.1%	7.9%	5.9%	8.5%	6.0%

### Prevalence of Blindness

83 (2.3% 95%CI 1.8–2.8) people were found to be blind (defined as visual acuity worse than 3/60 in the better eye with available correction). 41 (49.4%) were women. Table 2 shows the prevalence of blindness, severe visual impairment (visual acuity worse than 6/60 but equal or better to 3/60 in the better eye with available correction) and visual impairment (visual acuity worse than 6/18 but equal or better to 6/60 in the better eye with available correction) by gender. With best correction the prevalence of blindness decreased to 2.2% (80 people) (95%CI 1.6–2.8).

**Table 2:** Prevalence of Visual Loss by Gender.

	Male(n = 1,434)		Female (n = 2,195)		Total(n = 3,629)	
Visual Acuity	n	%	n	%	n	%
Blind with best correction *	39	2.72	41	1.87	80	2.2
**Blind with available correction (presenting)**†	42	2.93	41	1.87	83	2.29
**Severe Visual Impairment (presenting)**	29	2.02	34	1.55	63	1.74
**Visual Impairment (presenting)**	87	6.07	166	7.56	253	6.97
**Total**	158	11.02	241	10.98	399	11.00

### Causes of Blindness and Visual Impairment

Cataract was the main cause of blindness (39.8%) followed by posterior segment disease (34.9%). Overall 65.1% of blindness was avoidable. Table 3 shows the percentage of major causes by degree of visual loss. Causes such as glaucoma, age-related macular degeneration and diabetic retinopathy were grouped into posterior segment causes of visual loss because of diagnostic uncertainty. However, the survey suggested that approximately 47% of posterior segment disease was due to glaucoma.

**Table 3:** Degree and cause of presenting visual loss by person.

	Blind		SVI		VI		Total	
	n	%	n	%	n	%	n	%
**Cataract**	33	39.8	37	58.7	118	29.2	188	47.2
**Posterior Segment** *								
**Glaucoma**	25	30.1	8	12.7	4	1.6	37	9.3
**Diabetic Retinopathy**	0	0	0	0	3	1.2	3	0.75
**Age-related Macular Degeneration**	2	2.4	2	3.2	10	4	14	3.5
**Other**	2	2.4	3	4.8	16	6.3	21	5.3
**Total**	29	34.9	13	20.6	33	13	75	18.8
**Corneal Scarring**	12	14.5	7	11.1	22	8.7	41	10.3
**Phthysis**	5	6	0	0	0	0	5	1.3
**Refractive Error**	2	2.4	4	6.3	74	29.2	80	20.1
**Surgical Complications**	2	2.4	2	3.2	5	2	9	2.3
**Total**	83	100	63	100	252	100	398	100

* **Note: Posterior segment is presented overall and broken into component diseases.**

Cataract was the leading cause of severe visual impairment and visual impairment. The second largest cause of visual impairment was refractive error (29.2%).

### Cataract Surgery

86 people (113 eyes) had received cataract surgery at the time of the survey indicating a cataract surgical coverage of 46% (34% of eyes). Of all operated patients, 27 (0.7%) had received bilateral surgery.

26 (0.7%) people were bilaterally blind due to cataract, 148 (4.1%) people had one eye which was blind due to cataract. Table 4 summarises their answers when those who were bilaterally or unilaterally blind from cataract were asked why they had not had cataract surgery. 36.1% reported that they were unaware that any treatment was available for their condition.

**Table 4:** Reasons given for not having cataract surgery.

	Bilaterally blind fromcataract		Unilaterally blind from cataract		Total	
	n	%	n	%	n	%
**Unaware of treatment**	18	42.9	87	34.9	105	36.1
**Destiny/God’s will**	7	16.7	38	15.3	45	15.5
**How to get surgery**	2	4.8	33	13.3	35	12.0
**No services**	2	4.8	29	11.6	31	10.7
**Wait for maturity**	3	7.1	22	8.8	25	8.6
**No-one to accompany**	4	9.5	9	3.6	13	4.5
**Cannot afford**	2	4.8	9	3.6	11	3.8
**Contra-indication**	0	0	6	2.4	6	2.1
**One eye not blind**	0	0	5	2	5	1.7
**Old age: no need**	1	2.4	3	1.2	4	1.4
**Fear of operation**	2	4.8	2	0.8	4	1.4
**Fear of losing sight**	0	0	4	1.6	4	1.4
**No time**	1	2.4	2	0.8	3	1.0
**Total**	42	100	249	100	291	100

### Outcome of Cataract Surgery

**Figure 1:**
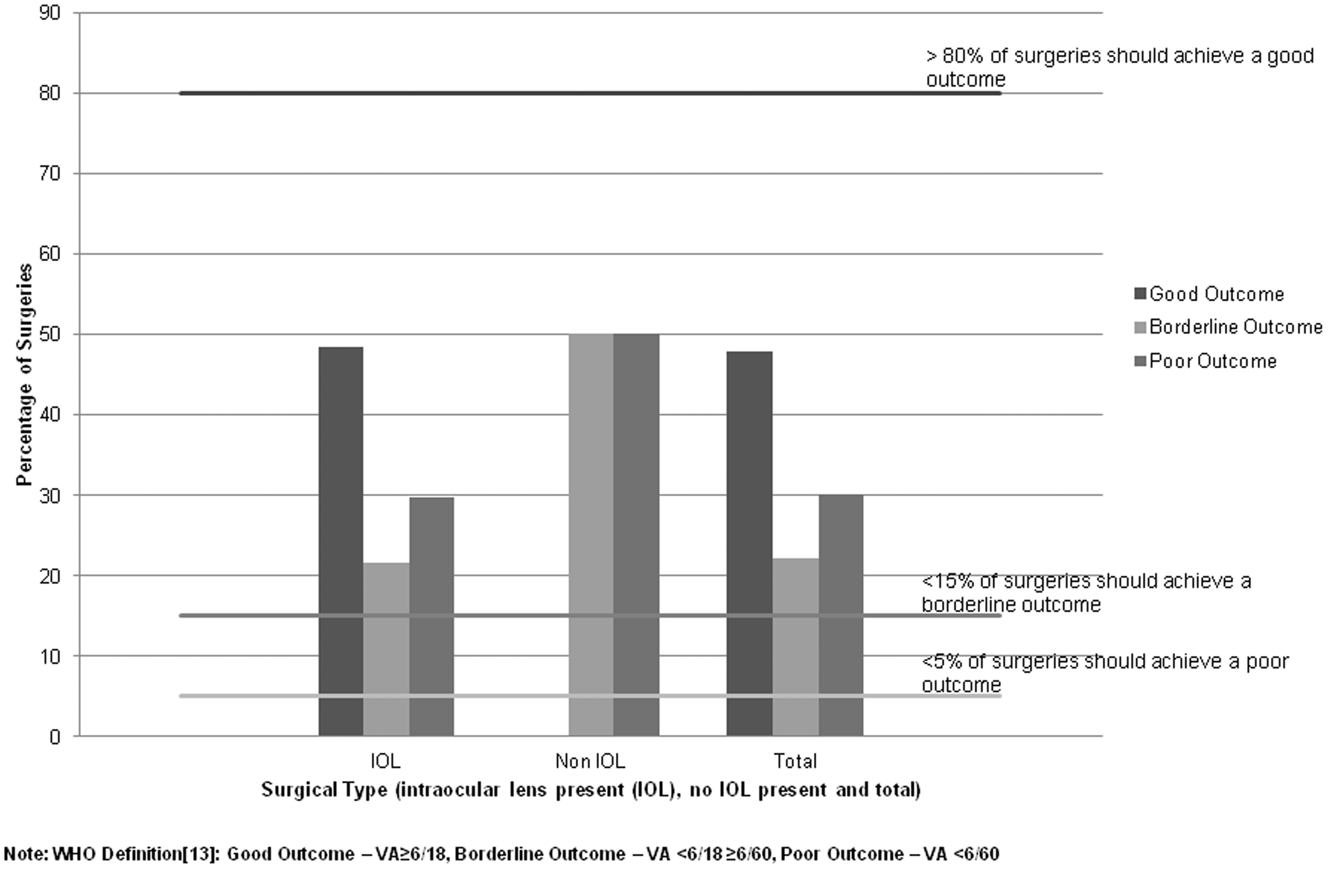
Percentage of poor outcomes with and without IOL insertion following cataract surgery in Southern Zambia compared to the WHO recommendations.

Of the 113 eyes that received cataract surgery at any time in the past; all except two had an intraocular lens (IOL) inserted. Figure 1 describes the outcome of surgery. 34 (30.1%) eyes had a presenting visual acuity of worse than 6/60 (termed ‘poor outcome’). The proportion decreased to 25.4% in those who had cataract surgery within 5 years of the survey date.

67 (59.2%) eyes had received cataract surgery in the five years preceding the survey. 17 (25.4%) eyes had a poor outcome and 36 (53.7%) had a good outcome, which increased to 40 (59.7%) with best correction.

The main causes of poor outcome after cataract surgery were co-morbidity (such as retinal disease or glaucoma) and complications during surgery.

When asked whether they were satisfied with the results of the surgery by eye, individuals reported they were very satisfied in 71% of surgeries and very unsatisfied in 15% of surgeries.

### Gender

Cataract surgical coverage (the proportion of those who are blind compared to those who have had surgery) suggests that women (65.0% coverage, 31.7% of operated eyes) were less likely to receive surgery than men (72.1% coverage, 40.9% of operated eyes). This is despite the fact that a similar proportion of men and women were blind from cataract.

### Extrapolation to the Survey Area

Assuming a prevalence of 2.29% and a population of 420,000 people over 50 in the survey area, it can be estimated that there were approximately 9600 people over 50 who were blind in the survey area at the time of the survey [12]. Approximately 4500 of these were blind due to cataract.

Based on the most recent WHO estimates, it is expected that 0.08% of the population under 15 years of age in Southern Province is blind [1]. The corresponding proportion in the age group between 15 and 49 is 0.16%^1^. Thus, approximately 13,300 people in all age groups in Southern Province were blind at the time of the survey, corresponding to an all age prevalence of 0.83%., with over 70% of cases occurring in people above 50 years of age [12].

## Discussion

This population-based survey established that approximately 2.3% of people in the area running along the railway line between Livingstone and Lusaka were blind. Over 65% of blindness was avoidable with the main cause being cataract, which affected approximately 8400 people over 50. More women than men were examined in the survey but this was a reflection of the population over 50 in the survey area.

The findings were similar to those of other RAAB surveys conducted in sub-saharan Africa where the prevalence of blindness ranged from 1.8% in Rwanda [6] to 3.3% in Malawi [4], a population with similar demographics to that in Zambia. The survey in Malawi reported cataract as the leading cause of blindness (48.2%). Posterior segment disease (reported as glaucoma) was found to be the second leading cause of blindness.

The survey in Malawi reported a very low number of cataract surgeries performed on women compared to this study. This was probably due to the predominantly rural location of the Malawi survey compared to the mixed urban-rural setting in this survey. However, the present survey also revealed a gender discrepancy between the proportion of men and women receiving cataract surgery in Zambia. Despite having a similar prevalence of cataract blindness, fewer women had received surgery than men. This difference has been found in different RAAB surveys[5]–[7], [14] in sub-Saharan Africa and remains an important issue for providers of cataract surgery who need to ensure their services reach everyone affected by disease.

This survey also found a high proportion of people who were blind from posterior segment disease (34.9%). The diagnosis was grouped in this way because it was made using a direct ophthalmoscope without any other tests, such as intraocular pressure measurement or bio-microscopy. Results from the survey suggest that the majority of posterior segment disease was due to glaucoma. This suggests that, with increasing cataract surgical services and, as a consequence, fewer people with cataract, diseases like glaucoma are becoming more prevalent causes of blindness.

The outcome of cataract surgery was suboptimal for many individuals, with over 30% of surgeries having a poor outcome despite an IOL being inserted. If surgeries are restricted to the five years before the survey, then the proportion of poor outcome decreases to 24%; however this still means that nearly one in four people do not see better than 6/60 despite having surgery. The main causes of poor outcome were co-morbidity and surgical complications. These results are similar to previous studies in sub-saharan Africa (including the survey in Malawi) and emphasise the importance of monitoring outcome and ongoing assessment of the quality of the services provided [4], [7], [15].

### Limitations of the Study

Precise, detailed examination was not possible as all diagnosis was made with a direct ophthalmoscope. This means that any posterior segment causes of visual loss should be interpreted with care. However, it was felt that the diagnosis was robust as agreement was tested prior to the survey beginning.

Care must be taken in interpreting the responses given by individuals to the question about why they had not had cataract surgery as this was not a rigorous qualitative assessment of the barriers to surgery and must only be used as a pointer to the main reasons why people did not take up surgery.

There was a marked discrepancy between the proportion of men and women examined in the survey. This reflects the population distribution of the country where there are a higher proportion of women than men over 50 years old and is similar to the situation in Malawi. However there was approximately equal representation between men and women who were blind. It is possible that blind men were over-represented in the sample because they remain at home whereas normal sighted men are able to leave the house and work.

### Conclusion

Blindness in the survey area between Lusaka and Livingstone affects approximately 1 in 50 of the population over 50. Nearly half is due to cataract – a treatable cause of blindness. The results from the RAAB Survey in Zambia provide important information to allow service providers, government and non-governmental organisations to plan services and advocate for eye care.

## Methods

### Ethical Approval

Ethical approval was granted by the Medical Research Ethics Committee of the University of Zambia.

### Timescale

The survey took place in June and July 2010.

### Setting

The survey area runs between Livingstone and Lusaka along a railway line and covers a population of approximately 3 million people, 12% of whom are aged above 50 [12].

An up-to-date age-specific list of the population per village was established using records from Central Statistical Office of Zambia in each district. This was thought to be the most reliable source of population data and was used as the sampling frame.

### Sample Size Calculation

The sample size was calculated using an estimated prevalence of 4%, with a variation of 20%, a non-compliance of 10%, a design effect of 1.5 and a 95% confidence limit. This was based on previous RAAB surveys in sub-Saharan Africa[4]–[6]. This gave a sample size of 3,830 people over the age of 50.

The sample size meant that 80 clusters (villages) were selected for the survey.

### Teams

Four teams were trained to conduct the survey. Each team consisted of an ophthalmologist or senior ophthalmic nurse and an assistant, either an ophthalmic clinical officer or an ophthalmic nurse.

### Training

Teams received one weeks training from an accredited RAAB trainer (RL) before starting the survey. Agreement between teams was assessed using a formal test where each team examined the same patient, assessing visual acuity and lens examination, and cause of any visual loss and reported their findings. Kappa was calculated and each team had to achieve at least 0.70 before the survey could commence.

### Selecting the Sample

There was a two stage sample selection. The first stage involved selecting villages from the sampling frame using probability proportional to size. The second stage consisted of dividing the village into segments of approximately 50 people over the age of 50. The segment of the village to be surveyed was selected using simple random sampling.

Each team moved from house to house in the selected segment. In each house every eligible person over the age of 50 was enumerated even if they were absent from the house. Eligibility was defined as residing in the house for more than 6 months in the previous twelve months, and sleeping in the house either the preceding or following nights. 50 people over the age of 50 were examined in each segment. If fewer than 50 people over the age of 50 were resident in the segment then a second segment from the cluster was selected by random and the survey continued until 50 people were found. Data collection lasted for six weeks.

If an eligible person was absent from the house at the time the survey team visited, then every effort was made to find and examine the individual. This included returning to the house at a later time, seeking the individual in another location or asking the individual to find the team when able. If it was not possible to examine the individual, family and friends were asked whether the person was blind.

Individuals who were eligible but for some reason unable to be examined (e.g. learning disabled) were recorded as unable to take part in the survey.

### Examination

Verbal consent was taken from each participant prior to any examination after a complete explanation of the study.

Each eligible individual had a visual acuity assessment using an illiterate tumbling E chart at six metres. Each eye was examined separately and the individual had to correctly identify four out of five characters on the E chart to achieve a specific category of visual acuity. ‘Presenting’ visual acuity assessment was conducted with their own distance spectacles if used. A pinhole visual acuity (termed ‘corrected’ visual acuity) assessment was conducted on all eyes that failed to see 6/18. Every individual then had an ocular examination using a direct ophthalmoscope and/or torch this included a lens assessment with the following categories; no/minimal lens opacity, significant lens opacity, pseudophakia with/without PCO or aphakia.Individuals whose visual acuity in either eye was worse than 6/18 with pinhole, then received, where appropriate, a dilated ocular examination by the ophthalmologist using a direct ophthalmoscope to determine the cause(s) of visual impairment.

### Data Collection

Data was collected using a standard proforma. This included information on visual acuity, the status of the lens and the causes of any visual loss. Causes of visual loss (worse than 6/18) were described for each eye and a main cause described for the person. The main cause of visual loss was determined by the ease of treatment, as specified by the WHO [13]. Hence, refractive error is easier to treat than cataract, so if an individual had both refractive error and cataract their main cause of visual loss would be refractive error. Blindness, severe visual impairment and visual impairment were defined according to the WHO categorization [13].

Information was also collected about any cataract surgery that had occurred or, for those with cataract, why cataract surgery had not taken place.

### Data Entry

Data was double entered into software developed for RAAB surveys. This allows automated entry cleaning and checking.

### Data Analysis

The RAAB software automatically produces reports on the results of the survey. This was supplemented by further analysis in Stata.

## References

[1] Pascolini D, Mariotti SP (2010). Global estimates of visual impairment.. Br J Ophthalmol doi.

[2] Pizzarello L, Abiose A, Ffytche T, Duerksen R, Thulasiraj R (2004). VISION 2020: The Right to Sight: a global initiative to eliminate avoidable blindness.. Arch Ophthalmol.

[3] WHO IAPB (2006). The VISION 2020 Action Plan, 2006-2011.. Geneva.

[4] Kalua K, Lindfield R, Mtupanyama M, Mtumodzi D, Msiska V (2011). Findings from a rapid assessment of avoidable blindness (RAAB) in Southern Malawi.. PLoS One.

[5] Mathenge W, Kuper H, Limburg H, Polack S, Onyango O (2007). Rapid assessment of avoidable blindness in Nakuru district, Kenya.. Ophthalmology.

[6] Mathenge W, Nkurikiye J, Limburg H, Kuper H (2007). Rapid assessment of avoidable blindness in Western Rwanda: blindness in a postconflict setting.. PLoS Med.

[7] Habiyakire C, Kabona G, Courtright P, Lewallen S (2010). Rapid assessment of avoidable blindness and cataract surgical services in Kilimanjaro region, Tanzania.. Ophthalmic Epidemiol.

[8] Kuper H, Polack S, Limburg H (2006). Rapid assessment of avoidable blindness.. Community Eye Health.

[9] UNDP (2011). National Human Development Reports for Zambia..

[10] Resnikoff S, Pascolini D, Etya’ale D, Kocur I, Pararajasegaram R (2004). Global data on visual impairment in the year 2002.. Bull World Health Organ.

[11] Zulu F (2005). A situation analysis of eye care services in Zambia.. Lusaka: Sightsavers International.

[12] Central Statistical Office (2003). Zambia 2000 Census of population and housing. Populations projections report.. Lusaka.

[13] WHO Programme for the Prevention of Blindness (1988). Coding instructions for the WHO/PBL eye examination record (version III).. World Health Organisation: Geneva.

[14] WHO (1998). Informal consultation on analysis of blindness prevention outcomes.. Geneva.

[15] Lindfield R, Kuper H, Polack S, Eusebio C, Mathenge W (2009). Outcome of cataract surgery at one year in Kenya, the Philippines and Bangladesh.. Br J Ophthalmol.

